# Activities of Daily Living, Functional Capacity, and Life Satisfaction  of Subacute Myelo-Optico-Neuropathy Patients in Japan

**DOI:** 10.2188/jea.JE20080085

**Published:** 2009-01-30

**Authors:** Tetsuya Kamei, Shuji Hashimoto, Miyuki Kawado, Rumi Seko, Takatoshi Ujihira, Masaaki Konagaya, Yukihiko Matsuoka

**Affiliations:** 1 Department of Medical Information Systems Course, Fujita Health University College, Toyoake, Aichi, Japan; 2 Department of Hygiene, Fujita Health University School of Medicine, Toyoake, Aichi, Japan; 3 Faculty of Nursing, Fujita Health University School of Health Sciences, Toyoake, Aichi, Japan; 4 Health and Welfare Bureau, Nagoya City, Nagoya, Japan; 5 Department of Neurology, Suzuka National Hospital, Suzuka, Mie, Japan; 6 Department of Neurology, Higashinagoya National Hospital, Nagoya, Japan

**Keywords:** subacute myelo-optico-neuropathy, SMON, activities of daily living, functional capacity, life satisfaction

## Abstract

**Background:**

Patients with subacute myelo-optico-neuropathy (SMON) suffer from a number of serious neurological symptoms that adversely affect their activities of daily living (ADL). However, the effects of these neurological symptoms on functional capacity and life satisfaction have not been reported.

**Methods:**

We analyzed data from 1,300 SMON patients aged 55–94 years that was obtained at medical check-ups carried out by the SMON Research Committee in 2004–2006 in Japan. The neurological symptoms investigated were visual impairment, dysbasia, symptoms of the lower extremities, and sensory symptoms. Neurological symptoms were classified by severity. The Barthel Index, the Tokyo Metropolitan Institute of Gerontology Index of Competence, and the participant’s response to the question “Are you satisfied with life?” were used to evaluate ADL, functional capacity, and life satisfaction, respectively. Data were analyzed using a proportional odds model with the scores for these items as ordinal dependent variables.

**Results:**

For most neurological symptoms, scores for ADL, functional capacity, and life satisfaction were significantly lower in participants with severe or moderate neurological symptoms than in those with nearly normal results upon examination. The odds ratio for life satisfaction due to superior functional capacity was significant after adjustment for sex, age, and ADL score.

**Conclusion:**

The presence of neurological symptoms in SMON patients was associated with low functional capacity, life satisfaction, and ADL. Our results suggest that the life satisfaction of SMON patients can be increased by improving their functional capacity.

## INTRODUCTION

Subacute myelo-optico-neuropathy (SMON) is a disease caused by clioquinol intoxication, and is characterized by subacute onset of sensory and motor disorders in the lower half of the body and visual impairment.^1^^,^^2^ In Japan, there are a large number of SMON patients.^3^ The incidence of SMON rapidly diminished after clioquinol was banned in 1970. In 2005, approximately 2,600 people with SMON were still receiving health management allowances as relief for an adverse drug reaction.^4^

Some studies have reported that a number of serious neurological symptoms have remained as sequelae of clioquinol intoxication among SMON patients, and that these symptoms are strongly associated with limited activities of daily living (ADL).^4^^–^^6^ However, both life satisfaction and functional capacity—which includes instrumental self-maintenance, intellectual activities, and social role—play an important role in the life of older SMON patients, and these are yet to be reported.^7^ In the present study, we examine the associations between neurological symptoms, ADL, functional capacity, and life satisfaction in SMON patients.

## PARTICIPANTS AND METHODS

### Participants

We analyzed data from medical check-ups performed by the SMON Research Committee with the support of the Ministry of Health, Labour and Welfare of Japan.^5^^,^^6^ Our study participants were SMON patients aged 55 to 94 years who underwent medical check-ups in the years 2004 to 2006. Of 1,326 participants, we excluded 11 who did not consent to the use of their medical check-up data for analysis, and 15 with missing data on ADL, functional capacity, or life satisfaction. Table 1 shows the number of participants eligible for analysis by sex and age. Of a total of 1,300 SMON patients (329 males and 971 females), 73% were between 65–84 years old.

**Table 1.** Number of participants included in analysis, by sex and age

Age	Men		Women		Total	
		
(years)	No.	%	No.	%	No.	%
55–64	39	11.9	138	14.2	177	13.6
65–74	152	46.2	318	32.7	470	36.2
75–84	115	35.0	366	37.7	481	37.0
85–94	23	7.0	149	15.3	172	13.2

Total	329	100.0	971	100.0	1,300	100.0

### Neurological symptoms

A standardized record was used in the medical check-ups for SMON patients.^5^^,^^6^ The record included visual impairment (completely blind, visual acuity insufficient to count fingers, mildly impaired, or nearly normal), dysbasia (abasia, clinging while walking, walking with a cane, or independent walking), symptoms of the lower extremities (severe, moderate, mild, or nearly normal), and sensory symptoms (severely diminished, moderately diminished, mildly diminished, or nearly normal). These levels were recorded as “severe,” “moderate,” “mild,” and “nearly normal,” respectively. Symptoms of the lower extremities were weakness, spasticity, and amyotrophy. Sensory symptoms were tactile sensation, algesthesia, vibratory sensation, and dysesthesia. Tactile sensation and algesthesia were divided into the 4 abovementioned levels plus “hypersensitive.”

### ADL, functional capacity, and life satisfaction

The Barthel Index was used to measure ADL.^8^ Scores ranged from 0 to 100, with a higher score denoting higher ADL. The Tokyo Metropolitan Institute of Gerontology Index of Competence (TMIG Index) was used to measure functional capacity^9^^,^^10^ and ranged from 0 to 13, with a higher score indicating higher capacity. Life satisfaction was evaluated using the response to the question, “Are you satisfied with life?” Participant responses were grouped into 5 categories: “dissatisfied,” “slightly dissatisfied,” “slightly satisfied,” “satisfied,” and “other.” We assigned scores of 1, 2, 4, 5 and 3, respectively, to these categories.

### Statistical analyses

ADL, functional capacity, and life satisfaction were compared among the severity groups for neurological symptoms by using a proportional odds model, which is a logistic model for ordinal dependent variables which assumes that the odds ratios for falling above a category versus those for falling within, or below, a category of ordinal dependent variables for independent variables are common across those categories.^11^^,^^12^ ADL, functional capacity, and life satisfaction were ordinal dependent variables. The model included sex, age, and one of the neurological symptoms (a dummy variable) as independent variables. The associations between ADL, functional capacity, and life satisfaction were examined using the proportional odds model with life satisfaction as an ordinal dependent variable and sex, age, ADL, and functional capacity as independent variables. Statistical analyses were conducted using SAS software, version 9.1 (SAS Institute, Inc., Cary, NC, USA).

### Ethical review

This study was approved in December 2005 by the Ethical Review Board for Epidemiological and Clinical Studies of the Fujita Health University School of Medicine.

## RESULTS

Table 2 shows the distributions of neurological symptoms. The proportions of participants with severe symptoms were 1.5% for visual impairment, 16.4% for dysbasia, 5.4–14.0% for symptoms of the lower extremities, and 10.1–35.3% for sensory symptoms. The proportions of participants with nearly normal results were 59.3%, 48.8%, 19.2–49.7%, and 3.0–4.3%, respectively.

**Table 2.** Distribution of neurological symptoms

Neurological symptoms	No.	Proportion (%)				

		Severe	Moderate	Mild	Hyper- sensitive	Nearly normal
Visual impairment	1247	1.5	6.8	32.4	-	59.3
Dysbasia	1249	16.4	9.7	25.1	-	48.8
Symptoms of lower extremities						
Weakness	1215	14.0	28.1	38.8	-	19.2
Spasticity	1212	7.1	16.9	26.3	-	49.7
Amyotrophy	1212	5.4	13.6	32.4	-	48.5
Sensory symptoms						
Tactile sensation	1211	10.2	41.5	34.5	9.4	4.3
Algesthesia	1211	10.1	34.3	28.3	23.1	4.2
Vibratory sensation	1205	35.3	35.4	24.9	-	4.4
Dysesthesia	1207	20.3	57.5	19.2	-	3.0

**Figure 1.**
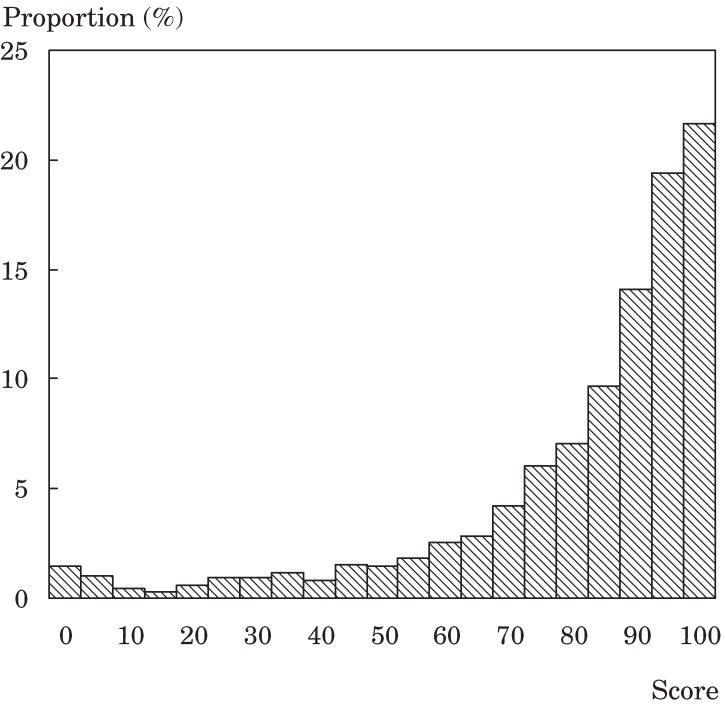
Distribution of activities of daily living scores

**Figure 2.**
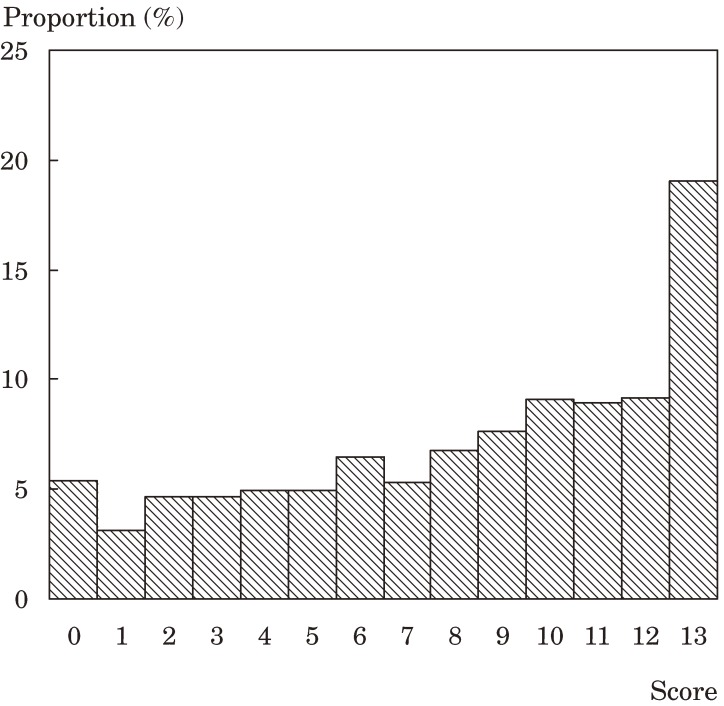
Distribution of functional capacity scores

**Figure 3.**
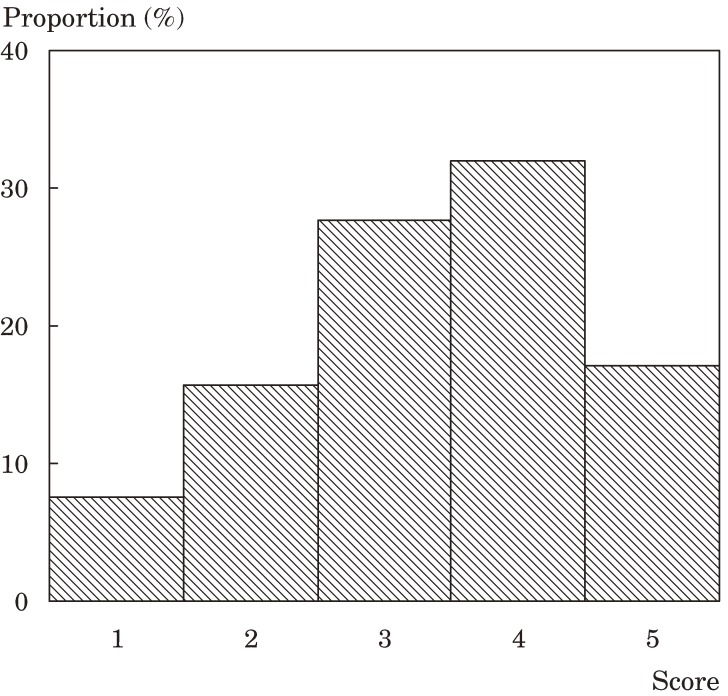
Distribution of life satisfaction scores

Figures 1, 2, and 3 show the distributions of the scores for ADL, functional capacity, and life satisfaction, respectively. The scores for ADL and functional capacity were widely distributed. For ADL, the proportion of participants scoring 70 or less was 22.1%. A functional capacity of 11 or less was noted in 71.8% of participants. With respect to life satisfaction, 23.2% of participants were “dissatisfied” or “slightly dissatisfied” and 49.1% were “slightly satisfied” or “satisfied”.

Tables 3, 4, and 5 show the respective mean scores and odds ratios for ADL, functional capacity, and life satisfaction for the groups of neurological symptoms. For all neurological symptoms, mean scores were lower in the severe and moderate groups than in the nearly normal group. For most neurological symptoms, after adjustment for sex and age, the differences in scores between the groups were statistically significant in analysis using the proportional odds model. The odds ratios for participants in the severe groups, as compared with the respective nearly normal group, were between 0.00 and 0.49.

**Table 3.** Mean scores and odds ratios for activities of daily living by severity of neurological symptoms

Neurological symptoms	Severe		Moderate		Mild		Hypersensitive		Nearly normal		*P* value†
				
	Mean score*	Odds ratio†	Mean score*	Odds ratio†	Mean score*	Odds ratio†	Mean score*	Odds ratio†	Mean score*	Odds ratio†	
Visual impairment	40.0	0.02	63.5	0.12	75.6	0.37	-	-	87.7	1.00	<0.001
Dysbasia	44.1	0.00	73.9	0.05	85.5	0.22	-	-	93.4	1.00	<0.001
Symptoms of lower extremities											
Weakness	49.2	0.01	77.6	0.13	89.5	0.45	-	-	94.5	1.00	<0.001
Spasticity	66.5	0.14	73.0	0.25	83.1	0.65	-	-	85.7	1.00	<0.001
Amyotrophy	38.9	0.02	65.4	0.09	81.9	0.39	-	-	90.6	1.00	<0.001
Sensory symptoms											
Tactile sensation	67.9	0.15	78.7	0.38	88.5	0.93	83.6	0.52	84.5	1.00	<0.001
Algesthesia	69.8	0.17	77.5	0.35	87.8	0.81	85.1	0.58	84.7	1.00	<0.001
Vibratory sensation	75.4	0.22	82.3	0.42	88.9	0.74	-	-	89.3	1.00	<0.001
Dysesthesia	72.6	0.24	82.6	0.57	88.8	1.18	-	-	81.7	1.00	<0.001

* Mean activities of daily living score.

† Odds ratio and *P* value from the proportional odds model with activities of daily living as an ordinal dependent variable and sex, age, and a neurological symptom as independent variables.

**Table 4.** Mean scores and odds ratios for functional capacity by severity of neurological symptoms

Neurological symptoms	Severe		Moderate		Mild		Hypersensitive		Nearly normal		*P* value†
				
	Mean score*	Odds ratio†	Mean score*	Odds ratio†	Mean score*	Odds ratio†	Mean score*	Odds ratio†	Mean score*	Odds ratio†	
Visual impairment	1.6	0.01	4.2	0.06	6.7	0.28	-	-	9.7	1.00	<0.001
Dysbasia	3.4	0.03	5.3	0.08	8.0	0.25	-	-	10.5	1.00	<0.001
Symptoms of lower extremities											
Weakness	3.8	0.04	7.2	0.17	9.3	0.48	-	-	10.9	1.00	<0.001
Spasticity	6.8	0.31	7.1	0.36	8.3	0.72	-	-	8.8	1.00	<0.001
Amyotrophy	2.7	0.03	5.5	0.13	7.8	0.38	-	-	9.9	1.00	<0.001
Sensory symptoms											
Tactile sensation	5.9	0.25	7.8	0.59	9.5	1.17	8.3	0.69	8.5	1.00	<0.001
Algesthesia	6.0	0.26	7.6	0.54	9.4	1.10	8.7	0.78	8.7	1.00	<0.001
Vibratory sensation	7.2	0.47	8.3	0.69	9.6	1.11	-	-	9.7	1.00	<0.001
Dysesthesia	7.0	0.35	8.3	0.60	9.4	0.95	-	-	8.7	1.00	<0.001

* Mean functional capacity score.

† Odds ratio and *P* value from the proportional odds model with functional capacity as an ordinal dependent variable and sex, age, and a neurological symptom as independent variables.

**Table 5.** Mean scores and odds ratios for life satisfaction by severity of neurological symptoms

Neurological symptoms	Severe		Moderate		Mild		Hypersensitive		Nearly normal		*P* value†
				
	Mean score*	Odds ratio†	Mean score*	Odds ratio†	Mean score*	Odds ratio†	Mean score*	Odds ratio†	Mean score*	Odds ratio†	
Visual impairment	2.2	0.16	3.0	0.46	3.2	0.66	-	-	3.5	1.00	<0.001
Dysbasia	3.1	0.42	3.2	0.47	3.2	0.56	-	-	3.5	1.00	<0.001
Symptoms of lower extremities											
Weakness	2.9	0.27	3.3	0.47	3.4	0.61	-	-	3.6	1.00	<0.001
Spasticity	3.1	0.63	3.2	0.68	3.2	0.67	-	-	3.5	1.00	0.002
Amyotrophy	2.9	0.33	3.1	0.53	3.3	0.67	-	-	3.5	1.00	<0.001
Sensory symptoms											
Tactile sensation	3.2	0.42	3.3	0.48	3.5	0.73	3.3	0.55	3.7	1.00	<0.001
Algesthesia	3.2	0.55	3.3	0.59	3.4	0.79	3.3	0.66	3.6	1.00	0.067
Vibratory sensation	3.3	0.47	3.3	0.45	3.4	0.53	-	-	3.8	1.00	0.021
Dysesthesia	3.2	0.49	3.3	0.54	3.6	0.89	-	-	3.7	1.00	<0.001

* Mean life satisfaction score.

† Odds ratio and *P* value from the proportional odds model with life satisfaction as an ordinal dependent variable and sex, age, and a neurological symptom as independent variables.

Table 6 shows the odds ratios for independent variables in the proportional odds model with life satisfaction as the ordinal dependent variable. The odds ratio for functional capacity—after adjustment for sex, age, and ADL—was significantly higher than 1.

**Table 6.** Odds ratios for life satisfaction with respect to sex, age, activities of daily living, and functional capacity

Independent variables	Odds ratio*	*P* value*
Sex (male/female)	1.51	<0.001
Age (years)	1.05	<0.001
Activities of daily living	1.01	0.109
Functional capacity	1.16	<0.001

* Odds ratio and *P* value from the proportional odds model, with life satisfaction as an ordinal dependent variable.

## DISCUSSION

We observed that the scores for ADL, functional capacity, and life satisfaction among SMON patients were strongly associated with the severity of neurological symptoms. The odds ratios of the severe group were much lower than those of the nearly normal group. These associations are not surprising, given that the neurological symptoms included visual impairment, dysbasia, symptoms of the lower extremities, and sensory symptoms. Some previous studies on ADL in SMON patients reported results similar to ours.^5^^,^^6^ We found that more than 70% of SMON patients had limited functional capacity (a score of ≤ 11 on the TMIG Index).^13^ Functional capacity includes instrumental self-maintenance, intellectual activities, and social role, and is likely to be an accurate indicator of the quality of life of older SMON patients.^10^^,^^13^ Our findings suggest that measures that can remedy the limited functional capacity of SMON patients are of great importance.^4^^,^^6^

When asked “Are you satisfied with life?” almost half of participants answered “slightly satisfied” or “satisfied.” Although interpreting these responses is difficult, the level of life satisfaction in SMON patients appears relatively low. A national survey in Japan reported that in the general elderly population the proportion of individuals who responded similarly to the same question was over 90%.^14^

In the present study, the level of life satisfaction was significantly associated with functional capacity after adjustment for sex, age, and ADL score, which suggests that life satisfaction among SMON patients might be increased by obtaining a higher level of functional capacity.^15^^,^^16^ Unfortunately, there are no effective medical treatments to relieve the remaining neurological symptoms in SMON patients.^4^^,^^5^ Although we did not investigate the factors associated with high functional capacity, our results offer information that should be useful in offering specific and effective assistance to those patients.

There are several limitations and problems in the present study. The participants were examined at medical check-ups carried out by the SMON Research Committee; approximately half of the participants were SMON patients receiving health management allowances for the relief of adverse drug reactions.^4^^,^^5^ Although the proportions SMON patients with limited ADL, functional capacity, and life satisfaction might be higher in the entire SMON population than in the subset of patients we analyzed, we believe that the associations between neurological symptoms and those indices would not be radically changed. The Barthel Index and TMIG Index used in this study are common tools for measuring ADL and functional capacity, respectively.^7^^–^^9^ We used the question “Are you satisfied with life?” to measure life satisfaction. Although other indices to measure life satisfaction have been proposed,^17^^,^^18^ questions similar to ours have been used in several previous studies.^14^^,^^19^ We used a proportional odds models for ordinal dependent variables of ADL, functional capacity, and life satisfaction, rather than binary logistic models that reduce those variables to just two categories. In proportional odds models, as we describe above, it is assumed that the odds of falling above a category versus those of falling with, or below, the category of ordinal dependent variables for independent variables are common across all categories.^11^^,^^12^ To take one example from the present study, the odds ratio for participants with severe visual impairment, as compared with those in the nearly normal group, was 0.49 for the dependent variable of ADL. This can be interpreted to mean that SMON patients with severe visual impairment had 0.49 times the odds of a higher, versus a lower, ADL score than those with nearly normal visual impairment. The results obtained from such models lead to important findings regarding the factors related to the distributions of ADL, functional capacity, and life satisfaction. However, their interpretation requires careful analysis and debate.

In conclusion, the neurological symptoms of SMON patients are associated with low levels of functional capacity, life satisfaction, and ADL. Our results indicate that life satisfaction of SMON patients might be increased by improving functional capacity.
